# Cluster Head Selection Method for Edge Computing WSN Based on Improved Sparrow Search Algorithm

**DOI:** 10.3390/s23177572

**Published:** 2023-08-31

**Authors:** Shaoming Qiu, Jiancheng Zhao, Xuecui Zhang, Ao Li, Yahui Wang, Fen Chen

**Affiliations:** 1 Communication and Network Laboratory, Dalian University, Dalian 116622, Chinachenfen@s.dlu.edu.cn (F.C.); 2 North Automatic Control Technology Institute, Taiyuan 030006, China

**Keywords:** routing protocol, edge computing, cluster head selection, Internet of Things

## Abstract

Sensor nodes are widely distributed in the Internet of Things and communicate with each other to form a wireless sensor network (WSN), which plays a vital role in people’s productivity and life. However, the energy of WSN nodes is limited, so this paper proposes a two-layer WSN system based on edge computing to solve the problems of high energy consumption and short life cycle of WSN data transmission and establishes wireless energy consumption and distance optimization models for sensor networks. Specifically, we propose the optimization objective of balancing load and distance factors. We adopt an improved sparrow search algorithm to evenly distribute sensor nodes in the system to reduce resource consumption, consumption, and network life. Through the simulation experiment, our method is illustrated, effectively reducing the network’s energy consumption by 26.8% and prolonging the network’s life cycle.

## 1. Introduction

In recent years, the rapid development of wireless network technology has made the Internet of Things (IoT) important in people’s productivity and life [1]. There is a wide range of sensor nodes distributed in the IoT, and they connect and communicate with each other to form a wireless sensor network (WSN), which plays an essential role in data collection and monitoring in the IoT [2,3]. However, with the rapid increase in devices connected to the network, cloud computing can no longer meet the low-latency computing requirements of these devices, and edge computing has been proposed [4]. Edge computing is a new type of network architecture. Because it is closer to the user end, it has low latency and high efficiency compared with cloud computing, and it also reduces bandwidth pressure [5,6]. At present, edge computing is widely used in WSNs. By using edge computing technology, it can effectively reduce the delay of WSN, improve the life of the network, and enhance the real time and reliability of the entire network system [7,8,9].

However, the energy of WSN nodes is still limited, and researchers have to consider designing some energy-saving routing protocols to reduce the energy consumption of WSN nodes [10]. By clustering WSN nodes, the energy consumption of nodes can be effectively reduced, and the network lifetime can be extended [11,12]. Usually, after the WSN nodes are clustered, a cluster head node is selected in each cluster to collect node data in the cluster and complete the transmission. Therefore, the quality of clusters and the selection of cluster heads will have a crucial impact on the performance and lifetime of the network [13,14].

In the work of this paper, to solve the problems of excessive energy consumption of nodes and short network life cycle caused by unreasonable clustering in the clustering routing protocol, we propose an optimization model of a two-layer WSN based on edge computing and an improved sparrow search algorithm is used to complete clustering. For the optimization problem, we use an improved sparrow search cluster head selection method, including chaotic initialization, sine–cosine mutation, and adaptive factor, to improve the network life cycle and wireless sensor network performance. The main contributions of this work are as follows:

(1) We propose a two-layer WSN system based on edge computing and establish an optimization model of a two-layer WSN based on edge computing;
(2) According to the optimization model of the two-layer WSN based on edge computing, we construct an optimization objective function to balance the load and distance factors in the clustering process;
(3) To solve the proposed problem, we adopt an improved sparrow search algorithm, which effectively improves the life cycle of the WSN.

The rest of this paper is organized as follows: Section 2 introduces the current research on clustering protocols for WSNs; Section 3 describes the system model and explains the problem model for clustering optimization in WSNs; Section 4 introduces the improved algorithm; Section 5 presents the simulation and comparison results; finally, Section 6 concludes the paper.

## 2. Related Works

Edge computing and WSNs have spread throughout people’s daily lives, such as environmental monitoring, smart grid, smart transportation, and intelligent agriculture [15,16]. The energy consumption of the WSN system can be effectively reduced through edge computing technology, and the network’s life can be extended. Just as [17] constructed an edge computing IoT architecture, the efficiency of calculation and the accuracy of calculation results are guaranteed through an adaptive hierarchical sampling method. Similarly, Ref. [18] proposes a task model in edge computing and a cluster-based heterogeneous WSN model and uses a task allocation algorithm that combines genetic algorithms and ant colony optimization algorithms to balance loads and reduce system energy consumption. The work in [19] aims at the problem that the traditional data cleaning method relying on sensor nodes affects cloud processing and proposes a data cleaning method based on mobile edge nodes, which can improve data cleaning efficiency while maintaining data reliability and integrity and effectively reduce energy consumption. The work in [20] designs an IoT-based cluster-based routing protocol and optimized cluster head selection, improving energy efficiency and network life. Similarly, Ref. [21] uses UAVs to assist the auxiliary wireless sensor network to complete the data collection work efficiently. The work uses drones as edge servers to effectively improve the efficiency of data collection. However, the energy of drones is limited and cannot complete data collection for a long time. Our edge servers mainly rely on base stations, which can assist in data collection for a long time.

Intelligent optimization algorithms have been widely used in the optimization of wireless routing protocols and the optimization of cluster head nodes [22,23,24,25]. The work in [13] uses a gray wolf optimization algorithm to select the best cluster head in WSN, saving energy and prolonging the network’s life. The work in [26] proposes a new ant colony WSN optimization strategy and designs a corresponding routing algorithm to extend the entire network’s life cycle by balancing each sensor node’s energy consumption. The work in [27] proposed an energy-saving cluster head selection algorithm based on particle swarm optimization, which considers the distance and remaining energy of nodes in the cluster and designs an efficient fitness function, which saves the energy of sensor nodes and extends the WSN lifetime. The fitness function described in [28] takes into account factors such as residual energy, distance to the sink, and node density to improve the selection of cluster heads. An improved genetic algorithm is proposed to select the best cluster head node, effectively improving network lifespan. The work in [29] proposes a WSN clustering routing algorithm based on a hybrid genetic tabu search. The cluster head selection process introduces node residual energy and node distance, effectively improving routing efficiency. The work in [30] proposed an energy-aware cluster routing protocol based on a seagull optimization algorithm for animal husbandry WSN, which can prolong the life of the network and reduce the end-to-end delay. The work in [31] initializes the population by selecting nodes in the monitoring area to increase the diversity of the population and uses the proposed optimization algorithm to choose a better cluster head set, which balances the energy consumption of sensor nodes, thereby prolonging the life cycle of the network.

Unlike the above studies, we propose a two-layer WSN system based on edge computing. The edge server can monitor the underlying WSN nodes and assist in the selection of cluster head nodes. Finally, the improved sparrow search algorithm (ICSSA-CHS) optimizes the selection of cluster heads, balances the energy of sensor nodes, and improves the life cycle of the network.

## 3. System Model

The two-layer WSN system based on edge computing mainly consists of an edge layer and a sensor node layer. In the edge layer are edge computing nodes, responsible for collecting relevant data information of sensor nodes, assisting cluster head selection, and transmitting sensor data. Generally, the cluster head node is not fixed and must be re-selected every round. When selecting the cluster head in each round, the nodes should be evenly distributed, and the load of the cluster head nodes should be balanced. After the cluster head selection is completed, ordinary nodes will join the nearest cluster according to the distance, and member nodes are assigned time slots in the cluster to complete data transmission. Nodes only transmit data in their assigned time slots and sleep in other time slots to reduce power consumption. At the end of each rotation period, the cluster head will be re-selected, and the new cluster head will re-select the cluster head according to the energy consumption.

We use $N=\{n_{1},n_{2},n_{3},\ldots ,n_{k}\}$ to denote all sensor nodes and all edge nodes are given by the set $S=\{s_{1},s_{2},s_{3},\ldots ,s_{k}\}$. The set $M=\{m_{1},m_{2},m_{3},\ldots ,m_{j}\}$ represents all clusters, we use $C_{q}=\left\{c_{1},c_{2},c_{3},\ldots ,c_{m}\right\},C_{q}\in M$ to represent the *q*-th cluster, and the cluster head node in the cluster is defined as $C_{q}^{h}$. When a node runs out of energy, we put it into the set *G*.

**Figure 1:**
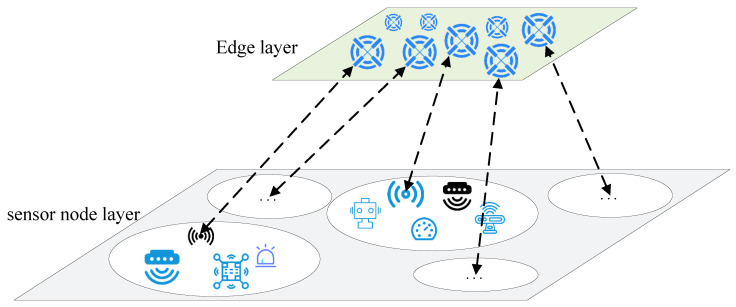
Edge network architecture.

Generally, in a WSN, once the corresponding sensors are arranged, they will not move anymore. In set $C_{i}$, all sensor nodes can communicate with each other and can communicate with edge nodes at the same time. Edge nodes can detect the remaining energy of different sensor nodes in the set. After the cluster head selection is completed, data transmission is mainly performed by the cluster head node and the edge node, as shown in Figure 1, which is the network structure diagram after the cluster head selection is completed. In the figure, all sensors at the sensor node layer may become cluster heads. The sensor node layer can represent a cluster formed in different areas, and the node pointed at by the arrow is defined as the cluster head. When cluster heads are re-selected, their regions may change. In the edge layer, some servers may be far apart geographically.

### 3.1. Node Energy Model

In the edge-side WSN, the sensor cannot be charged after the power is exhausted, so battery consumption is a crucial issue. Specifically, energy consumption in a WSN at the edge mainly comes from data sending and receiving. The energy consumption of the node data transmission process is mainly related to the size of the transmitted data packet and the transmission distance. The node data sending and receiving process satisfies the multipath fading channel and free-space model. A free-space model is typically used when the threshold distance exceeds the calculated distance. A multipath fading model is used in another case where the threshold is smaller [32]. The threshold distance $d_{0}$ is defined as

(1) $$d_{0}=\sqrt{\frac{\varepsilon_{fs}}{\varepsilon_{mp}}}$$

where $\varepsilon_{fs}$ is the energy required when using the free-space model, and $\varepsilon_{mp}$ is the energy of the power amplifier. Then its specific data transmission energy consumption formula is as follows:

(2) $$E_{tx}\left(k,d\right)=\left\{\begin{array}{c} k\;\;\;\cdot \;\;\;E_{n}+k\;\;\;\cdot \;\;\varepsilon_{\mathrm{fs}}\;\;\;\cdot \;\;\;d^{2},d<d_{0}\\ k\;\;\;\cdot \;\;\;E_{n}+k\;\;\;\cdot \;\;\varepsilon_{\mathrm{mp}}\;\;\;\cdot \;\;\;d^{4},d\geq d_{0} \end{array}\right.$$

where $E_{tx}\left(k,d\right)$ is the energy consumed by transmitting *k*-bit data for *d* meters, and $E_{n}$ is the energy consumption per unit of data sent or received by the sensor node. Different from sending data, the energy consumption when a node receives data is mainly related to the size of the data packet. Then the received data energy consumption $E_{r}x$ is defined as

(3) $$E_{rx}=k\times E_{n}$$

Therefore, the network communication energy consumption model of sensor nodes at the edge can be defined as

(4) $$E_{tot}=E_{tx}+E_{rx}+E_{0}$$

where $E_{tot}$ represents the total communication energy consumption of the node, and $E_{0}$ is the energy consumption of the node when it is dormant.

### 3.2. Node Residual Energy and Average Energy

Nodes in the edge-side WSN must ensure the life cycle of the nodes and the network during the cluster head selection process, so the remaining energy of the nodes needs to be considered during the selection process.

In the edge WSN, it is necessary to save the power of sensor nodes as much as possible to ensure the energy balance of sensor nodes and prolong the life of the network. Cluster head nodes need to consume more energy than ordinary nodes. Therefore, we need to select nodes that have consumed less energy as cluster head nodes as much as possible. The energy consumption $E_{i}^{r}$ of nodes is defined as

(5) $$E_{i}^{r}=\frac{E_{i}^{s}-E_{i}}{E_{i}^{s}}$$

where $E_{i}^{s}$ is the initial energy of the *i*-th sensor node; $E_{i}$ is the remaining energy of the current node of the *i*-th node. The smaller the value, the more abundant the energy of the cluster head node, the more suitable it is as the cluster head node.

In order to narrow the selection range of cluster head nodes and ensure that the cluster head nodes have a longer life cycle and can better assume the responsibility of data forwarding and aggregation, the edge layer nodes select the sensor nodes whose residual energy is greater than the average energy $E_{avg}$ of the selected nodes as candidate cluster head nodes and join the set of candidate nodes.

(6) $$E_{avg}=\frac{\sum_{i\in N\wedge i\notin G}E_{i}}{N-G}$$

where $E_{avg}$ is the average energy of all surviving nodes in the network, $E_{i}$ is the current remaining energy of sensor node *i*, and *G* is the number of dead nodes. When the remaining energy of the node is greater than $E_{avg}$, we put it into the set *H*.

### 3.3. Node Distance

In the edge-side WSN, the distances between nodes mainly include the distance between sensor nodes and edge nodes, the distance between cluster head nodes and common nodes, and the distance between cluster heads.

A Sensor node to edge node
  In the WSN at the edge end, the edge end needs to manage and monitor the sensor nodes, and the distance between them will affect the energy consumption of the nodes. Therefore, when selecting the cluster head, the node closer to the edge end should be selected as the cluster head node as much as possible. Specifically, the distance $D_{i}^{e}$ between the edge end and the *i*-th sensor node can be defined as
  (7) $$D_{i}^{e}=\frac{D_{i}}{D_{\max}}$$
  where $D_{i}$ is the distance between the *i*-th sensor node and the edge node, and $D_{max}$ is the distance between the furthest sensor node and the edge node.
B Inter-cluster node distance
  When selecting the cluster head nodes, ensure that the cluster head nodes in the network are evenly distributed as much as possible, so that the energy consumption of the cluster head nodes can be as uniform as possible, which can improve the network life cycle. Therefore, in the process of cluster head selection, the distribution of cluster head nodes and intra-cluster nodes needs to be considered. Specifically, we define the inter-cluster node distribution factor $D_{i}^{c}$. When it is smaller, the distribution of each intra-cluster node is more uniform. The specific definition is as follows:
  (8) $$D_{i}^{c}=\frac{{\sum_{i\in C_{q}}\left(C_{i}^{avg}-C_{avg}\right)}^{2}}{Q}$$
  where $C_{i}^{avg}$ represents the average number of neighbor nodes of all nodes in the *i*-th cluster, $C_{avg}$ represents the average number of neighbor nodes of *N* nodes in the entire network, and *Q* represents the total number of clusters.
C Distance between cluster heads
  In the process of cluster head selection, when the distance between cluster heads is large, the data need to go through more hops to reach the destination, which increases the delay of data transmission. Therefore, the distance between cluster heads is an important parameter in the cluster head selection algorithm, which determines whether the WSN can achieve the best routing performance. Therefore, the distance factor $D^{h}$ between cluster heads can be defined as
  (9) $$D^{h}=\frac{\sum_{i\in M}D_{i}^{e}}{\sum_{\forall i,j\in M}D_{i,j}^{h}}$$
  where $D_{i,j}^{h}$ represents the distance between cluster heads *i* and *j*, which is used to adjust the distance between cluster heads. $D_{i}^{e}$ represents the distance from the cluster head node *i* to the edge node. In order to make $D^{h}$ smaller, it is necessary to reduce the distance between cluster heads and edge nodes and, at the same time, increase the distance between cluster heads so that the distribution of cluster heads in the entire network is more dispersed.

### 3.4. The Optimization of the Target

In the cluster head selection process of the edge-side WSN, in order to balance the node energy and distance in the cluster head selection process, improve the distribution uniformity between clusters, and reduce the distance between cluster head nodes, our final optimization objective problem is expressed as

(10) $$\begin{array}{cc} \; & \min W=\theta_{1}\sum_{i\in H}E_{i}^{r}+\theta_{2}\sum_{i\in H}D_{i}^{e}+\theta_{3}\sum_{j\in C_{q},q\in M}D_{q,j}^{c}+\theta_{4}D^{h} \end{array}$$

(11) $$\begin{array}{cc} \;\;\;s.t. & C1\;\theta_{1}+\theta_{2}+\theta_{3}+\theta_{4}=1 \end{array}$$

(12) $$\begin{array}{ll} & \;\;\;C2\;0<\theta_{1},\theta_{2},\theta_{3},\theta_{4}<1 \end{array}$$

(13) $$\begin{array}{ll} & C3\;E_{i}\leq E_{i}^{s}\;\;\;\; \end{array}$$

where $\theta_{1}$, $\theta_{2}$, $\theta_{3}$, and $\theta_{4}$ are the weight factors, and *C*3 is the energy consumption constraint of the node.

## 4. Improved SSA Algorithm

To solve problem 10, we adopt an improved sparrow search algorithm. The sparrow search algorithm (SSA) is proposed by Xue et al. by simulating the foraging mechanism of sparrows [24,33]. To enhance the optimization efficiency of the SSA, we suggest using chaotic mapping and sine–cosine mutation. Additionally, we have introduced an adaptive population adjustment strategy. The following content will introduce the SSA and our improvement strategy.

### 4.1. The Sparrow Search Algorithm

The algorithm realizes optimization by simulating the three roles of producer, follower, and vigilant in the sparrow population. Among them, the producer is responsible for the global search for food-sufficient locations and provides foraging directions for followers. Generally, the producer in the sparrow population changes. Once a sparrow individual finds better food, it will be converted into a producer, but its proportion in the population is fixed. The producer’s location update can be defined as

(14) $$X_{i,j}^{t+1}=\left\{\begin{array}{c} X_{i,j}^{t}\cdot \exp\left(-\frac{i}{\alpha \cdot T_{\max}}\right),R<ST\\ X_{i,j}^{t}+Q\cdot L,R\geq ST \end{array}\right.$$

where $X_{i,j}^{t}$ is the *j*-th dimension of the *i*-th sparrow individual; *t* is the current number of iterations; $T_{max}$ indicates the maximum number of iterations; α is a random number that follows a uniform distribution within the range of (0,1]. *Q* is a random number that follows a normal distribution. The vector *L* is one-dimensional and has a length of *d*, with all elements set to 1. *R* is a uniformly distributed random number that represents the warning value of the population. ST represents the safety threshold of the population. Then, the follower’s position update can be defined as

(15) $$X_{i,j}^{t+1}=\left\{\begin{array}{cc} Q\cdot \exp\left(\frac{X_{worst}^{t}-X_{i,j}^{t}}{i^{2}}\right) & \;,i>n/2\\ X_{p}^{t+1}+\left|X_{i,j}^{t}-X_{p}^{t+1}\right|\cdot A^{+}\cdot L & \;,i\leq n/2\; \end{array}\right.$$

where $X_{worst}^{t}$ represents the global worst position in the current iteration; $X_{p}$ is the optimal position currently occupied by the producer; *A* is a one-dimensional vector whose elements are all 1 or −1, and $A^{+}=A^{T}{\left(AA^{T}\right)}^{-1}$. When i>2, the *i*-th follower sparrow receives very little food. In this case, the sparrow updates its position using the normal distribution law to search for more food. On the other hand, when i≤2, the individual sparrows jump to look for food near the finder. The vigilant’s location update can be defined as

(16) $$X_{i,j}^{t+1}=\left\{\begin{array}{c} X_{best}^{t}+\beta \cdot \left|X_{i,j}^{t}-X_{best}^{t}\right|\;\;,f_{i}>f_{g}\\ X_{i,j}^{t}+K\cdot \left(\frac{|X_{i,j}^{t}-X_{worst}^{t}|}{\left(f_{i}-f_{w}\right)+\varepsilon }\right)\;\;,f_{i}=f_{g} \end{array}\right.$$

where $f_{i}$ is the fitness value of the sparrow individual; $f_{g}$ is the current optimal fitness value; $f_{w}$ is the current worst fitness value; $X_{best}^{t}$ is the current global optimal position; β controls the step size and follows a standard random normal distribution; *K* is a random number in [−1, 1], used to control the moving direction of the sparrow; ϵ is a small constant that prevents the denominator from being zero. If $f_{i}>f_{g}$, the sparrow is at the outermost edge of the population and will move towards the optimal position. If $f_{i}=f_{g}$, the sparrow in the middle of the population has recognized the danger and will move to a different location.

### 4.2. Improved Strategy

To improve the optimization efficiency of the sparrow search algorithm, we propose a chaotic map to improve the initial distribution of the population; we introduce a sine–cosine mutation and an adaptive population adjustment strategy to improve the search performance.

#### 4.2.1. Improved Circle Chaos Initialization Population

To improve the efficiency of the sparrow search algorithm in the cluster head selection process, we redesigned the initialization method of the population. Specifically, we introduce a circle chaotic map to improve the initial population distribution so that the sparrow population can search the entire space more widely, thereby improving the optimization efficiency of the algorithm. The specific formula is as follows:

(17) $$X_{i,j}^{t}=\;\;\mathrm{mod}\;\;\left(3.845X_{i,j}^{t}-\left(\frac{0.69}{3.845\pi }\sin\left(3.84\pi \cdot X_{i,j}^{t}\right),1\right)\right.$$

where $X_{i,j}^{t}$ is the location of the *i*-th sparrow in the *j*-th dimension of the search space at the *t*-th iteration.

#### 4.2.2. Sine and Cosine Mutation Strategy

In order to improve the local search performance of the sparrow search algorithm, we introduce the sine–cosine mutation strategy. The sine–cosine algorithm can effectively improve the local search efficiency. Specifically, in the process of updating the position of the vigilante, we performed sine–cosine mutation on the position of the vigilante to improve the local search ability of the vigilante. The specific position update formula is as follows:

(18) $$X_{i}^{t+1}=\left\{\begin{array}{c} \left(X_{best}^{t}+\beta \cdot \left|X_{i}^{t}-X_{best}^{t}\right|\right)\cdot \sin r_{1}+\left(2\sin r_{1}\cdot \left|r_{2}X_{best}^{t}-X_{i}^{t}\right|\cdot \left(1-\frac{t}{T_{\max}}\right)\right)\;\;,\;f_{i}>f_{g}\\ \left(X_{i}^{t}+K\cdot \left(\frac{|X_{i}^{t}-X_{worst}^{t}|}{\left(f_{i}-f_{w}\right)+\varepsilon }\right)\right)\cdot \cos r_{1}+\left(2\cos r_{1}\cdot \left|r_{2}X_{best}^{t}-X_{i}^{t}\right|\cdot \left(1-\frac{t}{T_{\max}}\right)\right)\;\;,\;f_{i}=f_{g} \end{array}\right.$$

where $r_{1}$ is a random number in the interval [0,2π], and $r_{2}$ is a random number in the interval [0,2].

#### 4.2.3. Adaptive Population Adjustment Strategy

In the sparrow search algorithm, the number of producers is usually fixed. However, in the process of cluster head selection, if there are fewer producers in the early stage of the iteration, the search cannot be entirely performed; similarly, in the later stage of the iteration, there should be more followers to perform a local search to prevent falling into the local optimum untie. Therefore, we introduce adaptive parameters to adjust the number of producers and followers dynamically. The specific formula is as follows:

(19) $$\begin{array}{cc} \; & a=0.15\cdot \left(2e^{-\left(2t/T_{\max}\right)}-0.1k\right)+0.1\\ \; & P=a\cdot N\;\\ \; & S=(1-a)\cdot N \end{array}$$

where *N* is the total population, *k* is a random number in the [0, 1) interval, *P* is the number of producers, and *S* is the number of followers.

**Figure 2:**
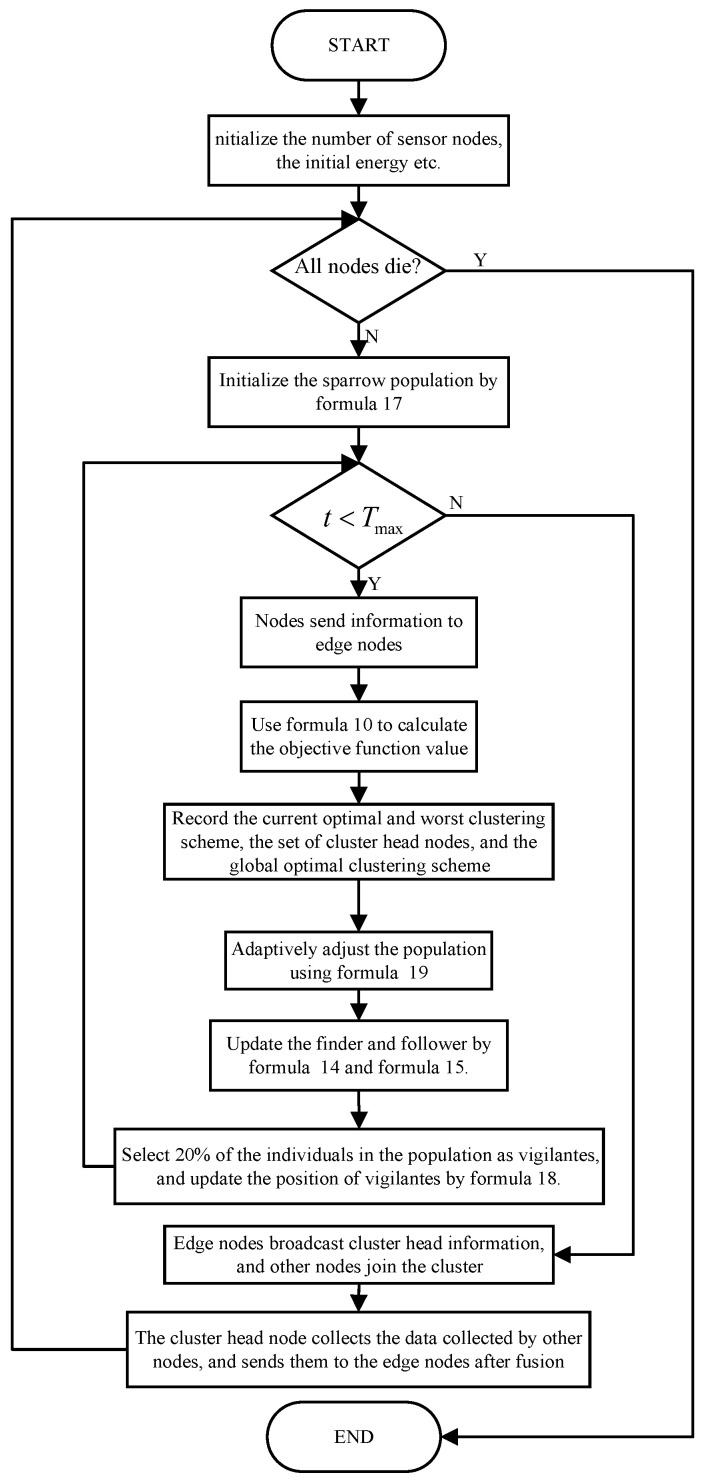
The flow chart of optimizing the clustering scheme of WSN based on ICSSA-CHS.

The specific steps of the flow chart of optimizing the clustering scheme of WSN based on ICSSA-CHS are as follows Figure 2, and in Algorithm 1 the pseudocode is provided.

Step 1: Initialize the number of sensor nodes, the initial energy, the size of the node distribution area, and use Formula (17) to initialize the sparrow population.

Step 2: Each node in the network sends its own position and energy information to the edge nodes. At this time, update the position and energy information of each node in the WSN at the edge node.

Step 3: If the maximum number of iterations is reached, go to step 6; otherwise go to step 4.

Step 4: Use Formula (10) to calculate the objective function value, and obtain the current optimal and worst clustering scheme, the cluster head node set, and the global optimal clustering scheme.

Step 5: Adaptively adjust the population according to Formula (19), select the currently optimal *P* sparrow individuals as discoverers and S sparrow individuals as followers, and update the discoverers and followers through Formulas (14) and (15).

Step 6: Randomly select 20% of individuals in the population as vigilantes, and update the position of the vigilantes through Formula (18), and execute step 3.

Step 7: Edge nodes broadcast cluster head information. After the cluster head node receives the information that it is selected as the cluster head node, it broadcasts to other nearby nodes, and other nodes join the cluster.

Step 8: The cluster head node collects the data collected by other nodes, and sends them to the edge nodes after fusion.

Step 9: Repeat until the lifetime of the WSN network ends Algorithm 1.
**Algorithm 1:** ICSSA-CHS
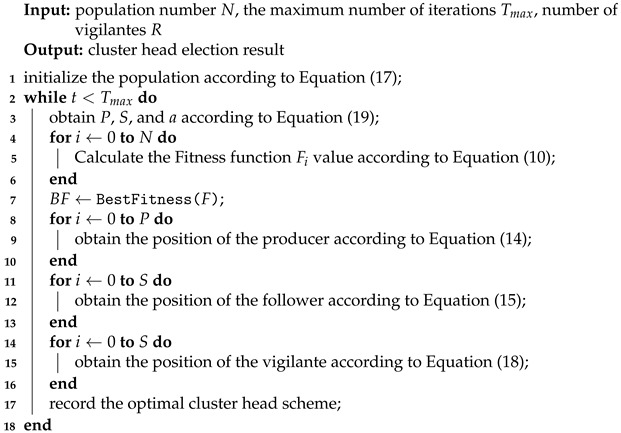


## 5. Simulation and Result

In this section, the proposed method is evaluated by evaluating the remaining energy consumption of nodes, the number of survivors, etc. Simulations were performed using Python on a computer with an Intel Core I7-7700HQ 2.8 GHz CPU and 16 GB RAM. The experimental parameter settings are shown in the Table 1.

**Figure 3:**
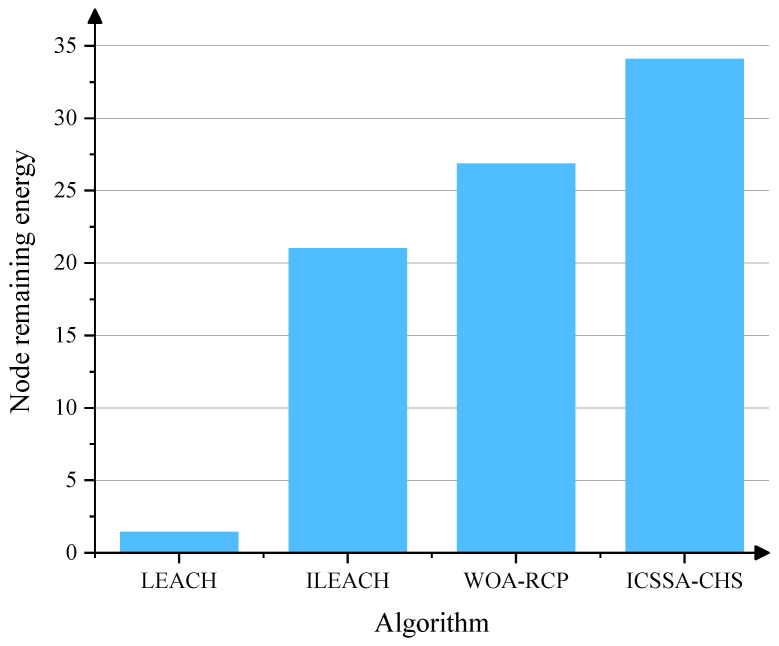
Node remaining energy after running 1000 rounds of different algorithms.

In order to verify the effectiveness of the improved sparrow search algorithm, we compared the improved cluster head selection method with LEACH, ILEACH, and WOA-RCP algorithms [31,34]. In the WSN, the remaining energy of nodes can effectively reflect the optimization of system energy consumption. As shown in Figure 3, there are 100 sensor network nodes in the monitoring area, and we analyze the remaining energy of our method and other methods after running 1000 rounds. Specifically, after running 1000 rounds, the remaining energy of our method is 34.08 *J*, which is 26.8% more energy than WOA-RCP.

**Figure 4:**
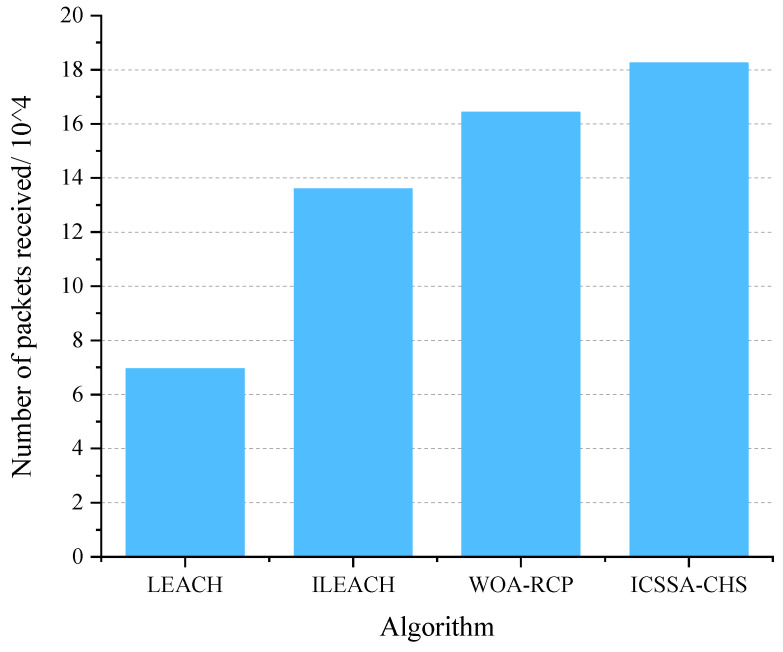
The number of data packets received by the edge end after running 2000 rounds of different algorithms.

Further, As shown in Figure 4, we compared the number of packets received at the edge end after running 2000 rounds. On the one hand, the more data packets the edge end receives, the more stable the system network transmission will be. On the other hand, it fully demonstrates that the ICSSA-CHS algorithm can effectively improve the network life cycle. Our system has a stronger data transmission capability when the edge end receives more data packets. The number of packets transmitted by our algorithm is $18.25\times 10^{4}$, which is 11.2% higher than WOA-RCP.

**Figure 5:**
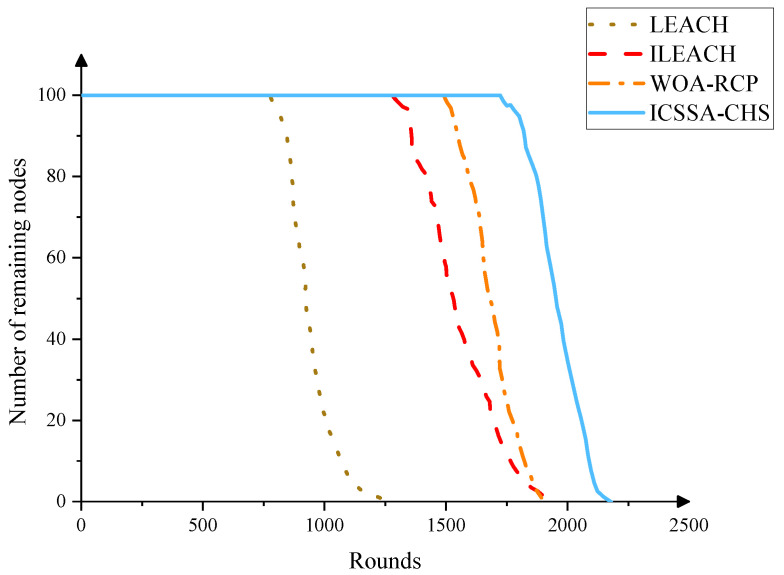
The number of remaining nodes of different algorithms after the number of running rounds increases.

We also analyze how the remaining surviving nodes change when the number of running rounds increases. Generally, the more running rounds and remaining nodes, the stronger the life cycle and data transmission capability of the network. As shown in Figure 5, as the number of running rounds increases, the number of generated nodes begins to decline after remaining unchanged. This is because as the number of running rounds increases, and the energy of some nodes is exhausted. The original LEACH protocol first dropped to 0. We introduce edge nodes through a two-layer network structure, reducing the burden on the cluster head nodes and energy consumption speed. At the same time, as the control node, the edge node uniformly adjusts and allocates the cluster head node and member nodes during data fusion and transmission, saving energy consumption in the cluster and improving the network life. Ultimately, our proposed method sustains the network lifespan over 2000 rounds.

**Figure 6:**
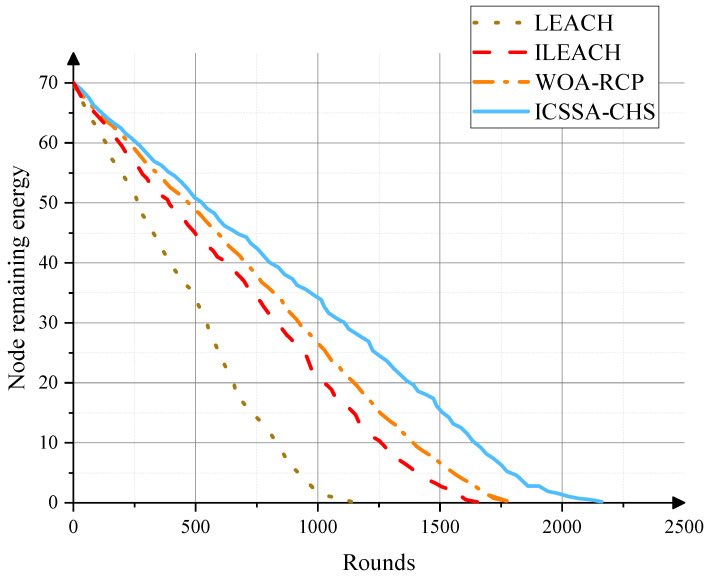
The remaining energy of nodes of different algorithms after the number of running rounds increases.

In addition, we analyze the relationship between the number of running rounds and the remaining energy of network nodes. Usually, the more energy left in each round, the lower the energy consumption of the node during the transmission process, which shows that our method can effectively improve the life of the network. As shown in Figure 6, it can be observed that as the number of rounds increases, the energy of network nodes decreases. This is because of the energy consumption caused by node operation. However, compared with other protocols, the ICSSA-CHS protocol performs well in reducing the remaining total energy of nodes, and the energy consumption of each round of the network is also less, making the node run up to 2206 rounds, which is higher than the other three methods and the limited extension of the network life cycle.

**Figure 7:**
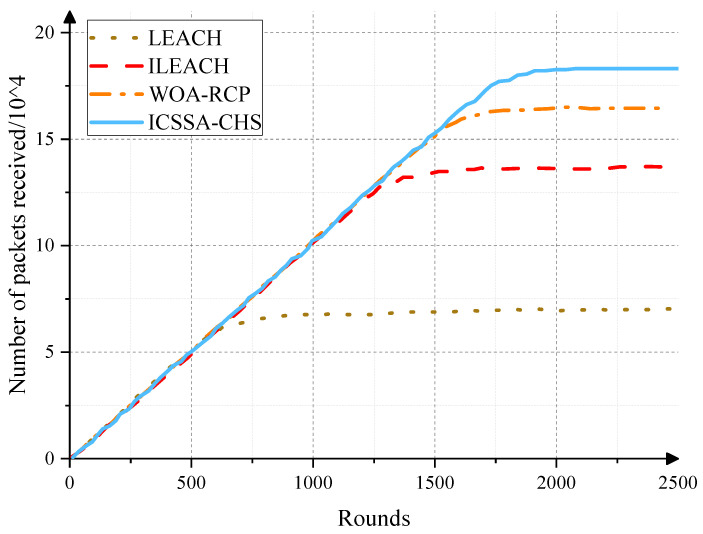
The number of edge-end data packets received by different algorithms after the number of running rounds increases.

In addition, we analyze the number of packets received by edge nodes, which is used to evaluate the information transmission capacity of the network. As shown in Figure 7, as the number of running rounds increases, the number of packets received by edge-end nodes increases gradually. This is because, with the operation of the network, the data exchanged by the sensor nodes gradually increase. However, the CMASSA-CHS protocol shows a higher level of data packet transmission than the LEACH, ILEACH, and WOA-RCP protocols, which shows that the CMASSA-CHS algorithm is more reasonable in selecting cluster heads and network clustering, which makes network transmission more stable and extends the network life cycle.

## 6. Conclusions

WSN plays an essential role in the data collection of the IOT. To reduce the energy consumption of sensor nodes and prolong the network’s life, this paper constructs a two-layer protocol structure system model of WSN. Then, we design an optimization problem that balances distance and energy consumption based on the proposed model. Finally, we optimize the cluster head selection through the improved sparrow search algorithm, including chaotic initialization, sine–cosine mutation, and adaptive population adjustment strategy. This effectively reduces node energy consumption, improves network performance, and prolongs the network life cycle. Specifically, compared with WOA-RCP by the ICSSA-CHS algorithm, we reduce energy consumption by 26.8%, improve network packet transmission, and prolong the network lifetime. In the future, we will further study issues such as node data collection and fusion compression under the double-layer WSN structure to improve network efficiency.

## Figures and Tables

**Table 1:** Average optimization result.

Parameter	Value
Experimental area	150×150
The number of sensor network nodes	100
Number of edge nodes	10
packet size/bit	4000
The maximum number of running rounds of the network	2500
Node initialization energy/J	0.7
Fusion Data Consumption/$J\cdot bit^{-1}\cdot packet^{-1}$	0.01
$E_{n}/nJ\cdot bit^{-1}$	50
$\epsilon_{fs}/pJ\cdot bit^{-1}\cdot m^{-2}$	10
$\epsilon_{mp}/pJ\cdot bit^{-1}\cdot m^{-2}$	0.0013
Population size	100
Iterations	300
Ratio of vigilantes	0.2

## Data Availability

Not applicable.
